# Effect of cyclical intermittent hypoxia on Ad5CMVCre induced solitary lung cancer progression and spontaneous metastases in the Kras^G12D+^; p53^fl/fl^; myristolated p110^fl/fl^ ROSA-gfp mouse

**DOI:** 10.1371/journal.pone.0212930

**Published:** 2019-02-27

**Authors:** Xiaofeng Guo, Yan Liu, Jessica L. Kim, Emily Y. Kim, Edison Q. Kim, Alexandria Jansen, Katherine Li, May Chan, Brendan T. Keenan, Jose Conejo-Garcia, Diane C. Lim

**Affiliations:** 1 Center for Sleep and Circadian Neurobiology, University of Pennsylvania, Philadelphia, Pennsylvania, United States of America; 2 Department of Otolaryngology Head and Neck Surgery, the Second Hospital, Jilin University, Changchun, Jilin Province, China; 3 Department of Immunology, H. Lee Moffitt Cancer Center & Research Institute, Tampa, Florida, United States of America; 4 Division of Sleep Medicine, Department of Medicine, University of Pennsylvania, Philadelphia, Pennsylvania, United States of America; University of South Alabama Mitchell Cancer Institute, UNITED STATES

## Abstract

**Background:**

Epidemiological data suggests that obstructive sleep apnea (OSA) is associated with increased cancer incidence and mortality. We investigate the effects of cyclical intermittent hypoxia (CIH), akin to the underlying pathophysiology of OSA, on lung cancer progression and metastatic profile in a mouse model.

**Methods:**

Intrathoracic injection of Ad5CMVCre virus into a genetically engineered mouse (GEM) Kras^G12D+/-^; p53^fl/fl^; myristolated-p110α^fl/fl^-ROSA-gfp was utilized to induce a solitary lung cancer. Male mice were then exposed to either CIH or Sham for 40–41 days until harvest. To monitor malignant progression, serial micro CT scans with respiratory gating (no contrast) was performed. To detect spontaneous metastases in distant organs, H&E and immunohistochemistry were performed.

**Results:**

Eighty-eight percent of injected Ad5CMVCre virus was recovered from left lung tissue, indicating reliable and accurate injections. Serial micro CT demonstrated that CIH increases primary lung tumor volume progression compared to Sham on days 33 (p = 0.004) and 40 (p<0.001) post-injection. In addition, CIH increases variability in tumor volume on day 19 (p<0.0001), day 26 (p<0.0001), day 33 (p = 0.025) and day 40 (p = 0.004). Finally, metastases are frequently detected in heart, mediastinal lymph nodes, and right lung using H&E and immunohistochemistry.

**Conclusions:**

Using a GEM mouse model of metastatic lung cancer, we report that male mice with solitary lung cancer have accelerated malignant progression and increased variability in tumor growth when exposed to cyclical intermittent hypoxia. Our results indicate that cyclical intermittent hypoxia is a pathogenic factor in non-small cell lung cancer that promotes the more rapid growth of developing tumors.

## Introduction

Obstructive sleep apnea (OSA) is an increasingly common sleep disorder [1], with significant morbidity and mortality [2]. While some studies associate OSA with an increased incidence [3, 4] and mortality from cancer [5–7], other studies do not show this association [8, 9]. It may be that different elements of OSA, such as severity of sleep fragmentation and cyclical intermittent hypoxia (CIH), affect tumor progression differently [2, 10, 11]. This argues that pre-clinical studies are needed to investigate specific mechanisms. Both CIH [12] and sleep fragmentation [13] can be produced in mouse models. In the study reported here, we investigate the effects of CIH.

It has been proposed that CIH promotes cancer by stabilizing HIF1α [14] thereby increasing cell proliferation and tumor angiogenesis [15], evading immune surveillance [16] and activating the PI3K signaling pathway [17], as well as induce changes in oxidative stress, metabolism, autonomic nervous system, and hormonal balance [16]. While CIH studies that utilize subcutaneous cancer models (injection of lung cancer cells into the flank of a mouse) [16, 18, 19] have significantly contributed to our understanding of CIH on cancer, no CIH studies have been performed using a genetically engineered mouse (GEM) model of lung cancer, which may be a more relevant replication of human disease. While subcutaneous lung cancer models are easy to track and study, Graves et al [20] demonstrated that this model is significantly more hypoxic than orthotopic lung cancer models (injection of lung cancer cells into the lung of a mouse), thus a GEM model of lung cancer may have clinical relevance.

Lung cancer is the leading cause of cancer-related deaths worldwide, with a poor prognosis [21] despite significant advances in understanding the molecular events leading to progression and metastasis [22, 23]. Important to this understanding is the mouse model of lung cancer, which has undergone a significant evolution over the past 20 years. The two most common mutations found in human lung cancer, Kras and p53 [24, 25], are widely used in a GEM model of lung cancer. Recently, the addition of the myristolated-p110α^fl/fl^ mutation produces an accelerated lung tumor progression [17]. While intranasal inhalation and intratracheal instillation of Ad5CMVCre virus in double and triple transgenics are the most common method of delivery to induce cancer, this method turns on multiple lung and airway adenomas, of which some of them progress to a carcinoma, which is not how cancer initiates or progresses in humans with lung cancer. Herter-Sprie [26] recently described an intrathoracic injection method that produces a single lung tumor which may be more clinically relevant because a single lung tumor is the premise of staging lung cancer in humans, with any second lung tumor (ipsi- or contralateral) considered to already be metastatic [27] with a poor prognosis.

In the current study, we report the effects of CIH on lung cancer progression and evidence of metastases in a triple transgenic GEM model, *Kras*^*G12D+/-*^; *p53*^*fl/fl*^; *myristolated-p110α*^*fl/fl*^*-ROSA-gfp*, developed by Sheen et al [17]. Confirming our hypothesis, we found that CIH accelerated primary lung tumor volume progression. In addition, we report the presence of metastases of the lung tumor to right lung, mediastinal lymph nodes, heart and ribs at the time of sacrifice, 40–41 days after induction of the lung tumor. Thus, we show that the GEM model *Kras*^*G12D+/-*^; *p53*^*fl/fl*^; *myristolated-p110α*^*fl/fl*^*-ROSA-gfp* injected with Ad5CMVCre virus develops a solitary lung tumor that spontaneously metastasizes. Moreover, we demonstrate that compared to sham animals, cyclical intermittent hypoxia results in increased tumor progression and tumor variability.

## Methods

The animal study protocol was reviewed and approved by the institutional animal care and use committee of the Perlman School of Medicine at the University of Pennsylvania. In combining several established approaches to induce lung cancer into one model, we first assessed the reproducibility of the intrathoracic injection technique by measuring the amount of Ad5CMVCre virus recovered in the lung (n = 14) immediately after injection, and in separate animals (n = 3), we confirmed gfp activation in the left lung 4 days after Ad5CMVCre virus injection. To assess the effect of CIH on lung cancer, we exposed mice to CIH (n = 18) or Sham (n = 17) for 2 weeks, injected mice with Ad5CMVCre virus to initiate cancer, then continued to expose mice to CIH or Sham until harvest. We assessed tumor volume progression using micro computed tomography (CT) at weekly time points following tumor initiation in mice exposed to sham conditions and those undergoing cyclical intermittent hypoxia (CIH).

### Transgenic mice and cyclical intermittent hypoxia

#### Transgenic mice

Transgenic *Kras*^tm4Tyj^ and *Trp53*^tm1Brn^ mice [28, 29] were obtained from NCI Mouse Models of Human Cancers Consortium, brought to a full C57BL/6 background [30], and bred with a C57BL/6 Cre-inducible myristoylated-p110α*-ROSA-gfp* mice [31, 32]. Conditional mutant mice were bred and genotyped using PCR as previously described [17, 31]. All mice were homozygous for p53^fl/fl^ deletion and myristoylated-p110α^fl/fl^*-ROSA-gfp* and, as a proof-of-concept study, only male Kras^G12D+/-^ mice were used.

#### Cyclical intermittent hypoxia

At 2–3 months old, 35 male mice were placed into the Hycon (Slava Savransky, West Des Moines, IA) and exposed to CIH (n = 18) or Sham (n = 17). Details of the Hycon are described in a prior publication [12]. In short, in the CIH group, mice were exposed to decreasing FiO_2_ over 45 seconds [from room air (FiO_2_ = 0.21) to an oxygen saturation (SaO_2_) nadir of 39% (FiO_2_ = 0.06)], then back to room air over 15 seconds. Mice were exposed to cyclical intermittent hypoxia (CIH) or room air (sham) for 12 hours per day during lights on (7AM-7PM) for 2 weeks, and then all mice underwent intrathoracic injection with Ad5CMVCre virus. After injection of Ad5CMVCre virus, mice were allowed to recover for 24 hours, then placed back into Hycon (CIH or Sham) for a total of 40–41 days, until the time of harvest (Day 40–41). Beginning on day 12 after injection, micro CT scans were performed at weekly intervals (on days 12, 19, 26, 33 and 40). Mice were removed from the Hycon for approximately 6 hours (during lights on) to perform scans.

#### Mouse survival and monitoring

Using the paradigm described above, all mice were scheduled to be sacrificed on Day 41 post-injection, unless per protocol earlier sacrifice was required due to observable distress. To monitor for signs of distress (respiratory distress, decreased movement, decreased body weight), mice were monitored daily. For this experiment, among **CIH mice (total n = 18)**: 1 mouse had all 5 scans but died before harvest (no tissue collection), 1 mouse had 4 scans and died before 5^th^ scan (4 uCT scans were included in analyses and no tissue collected), and 7 mice did not develop tumor (uCT scans were not included in analyses because there were no lung tumors most likely due to suboptimal injection), so final CT scan analyses was performed on 11 CIH mice and harvest was performed in 9 CIH mice. For **Sham mice (total n = 17)**: 1 mouse had all 5 scans but died before harvest (no tissue collection), no mice died before final scan and 4 mice did not develop tumor (CT scans not analyzed because there were no lung tumors most likely due to suboptimal injection), so final CT scan analyses was performed on 13 Sham mice and harvest was performed in 12 Sham mice.

### Ad5CMVCre virus intrathoracic injection: Validation of injection and Cre activation

#### Ad5CMVCre virus

Ad5CMVCre virus (Gene Transfer Vector Core, University of Iowa) was precipitated at a concentration of 3.12x10^7^ plaque forming units (pfu)/3uL according to manufacture guidelines. Prior to each injection, the virus was mixed before drawing up Ad5CMVCre virus using a Hamilton syringe (33G needle).

#### Intrathoracic injections

Injections were always done in the left lung between the 9^th^ and 10^th^ rib. Under 2% isoflurane, the mice were given SQ buprenorphine 0.5mg/kg XR and meloxicam 5mg/kg and then placed in the right lateral decubitus position (right side down, left side up). After fur was shaved and cleansed with alternating 70% alcohol and 20% chlorhexidine, a visual axis was determined using landmarks from the back of the left ear to the bottom of the left shoulder blade. A 1-2cm incision was made along this axis below the left shoulder blade. The fat pad was dissected until the lung was visualized. Coordinates of the injection were 10mm from the posterior edge of the lung, 5mm from the left lateral edge of the lung and 3mm deep. Using a Hamilton syringe, 3.12x10^7^ pfu/3μL activated Ad5CMVCre solution was injected directly into the lung. Tissue adhesive (LiquiVet, 8 second, SKU: VG3SC, Vendor: Oasis, ShopMedVet.com) was used to close the incision.

#### Validation of intrathoracic injection

Variability of Ad5CMVCre virus injection was accessed by a customized real-time PCR assay. The TaqMan primers (For: 5’-gcttatatagacctcccaccgtacac-3’; Rev: 5’-cgtggatagcggtttgactcac-3’) and probe (FAM-MGB; 5’-ccattgacgcaaatgggcggtaggc-3’) were designed to target a CMV promoter region that was specific to the virus. The assay was performed using a modified method adapted from prior studies [33, 34]. After mice were injected, lung tissue was dissected within 5 minutes and immediately snap frozen in liquid nitrogen. For DNA extraction, 1mL of lysis buffer (50mM Tris-HCl [pH8.5], 1mM EDTA, 0.5% SDS, 200μg/mL Proteinase K) was added directly to lung tissue and incubated overnight (~16hr) at 55°C with shaking (250rpm). Proteinase K activity was inactivated by 10 minutes of incubation at 95°C, and after 2 minutes of centrifugation at 20,000g, 400μL of supernatant was transferred to a clean tube. We then used TaqMan RT-PCR reactions on the crude DNA extract to quantitate the amount of Ad5CMVCre virus recovered from the lung of each mouse (n = 14) injected with Ad5CMVCre by comparing the measured Ct values to a standard calibration curve.

The standard calibration curve of Ct values versus pfu was developed by using freshly dissected lung tissues from three wild-type mice that was then spiked with increasing known quantities of Ad5CMVCre virus, each into a separate Eppendorf tube. First, a blank sample was made using wild-type lung tissue and no Cre virus. The three lowest concentrations of the standard calibration curve were made by mixing a 3.12x10^6^ pfu spiked lung sample with a blank sample at a 1:9, 1:4, and 1:1 ratio, resulting in samples containing equivalent amounts of 3.12x10^5^, 6.24x10^5^, and 1.56x10^6^ pfu of Ad5CMVCre virus, respectively. The highest three concentrations of the standard calibration curve were made by spiking in 3.12x10^6^, 1.56x10^7^, and 3.12x10^7^ pfu of Ad5CMVCre virus into a blank sample. DNA extractions were performed on each sample of the calibration curve as described above. RT-PCR was performed on all 6 standard calibration samples and 14 lung samples from mice injected with Ad5CMVCre using an Applied Biosystems 7500 system real-time PCR machine with a 10μL reaction containing 2μL of 20x dilution of the crude DNA and 2% Tween 20.

#### Validation of Ad5CMVCre virus activity within the left lung

As previously described, 3.12x10^7^ plaque forming units (pfu)/3uL of Ad5CMVCre virus was injected into left lung of *Kras*^*G12D+/-*^; *p53*^*fl/fl*^; *myristolated-p110α*^*fl/fl*^*-ROSA-gfp* mice (n = 3). As a negative control, a *Kras*^*G12D+/-*^; *p53*^*fl/fl*^; *myristolated-p110α*^*fl/fl*^*-ROSA-gfp* mouse (n = 1) was not injected with Ad5CMVCre virus. 4 days after the Ad5CMVCre virus injection, mice were harvested to assess virus activation (for details of harvest, see “Histological assessment”). The 4-day time point was chosen as this would be too early for metastases to occur and a previous study demonstrated Adeno-Cre activation as early as 3 days [35]. A cryostat was used to slice tissue at 8um and immunohistochemistry for gfp (Life Technologies, G10362) was performed to localize Ad5CMVCre activity within the left lung. Micrographs were obtained using a 1.4-megapixel CCD camera (DFC– 360FX, Leica, Germany) mounted on a digital microscope (DM5500B, Leica, Germany).

### Serial micro computed tomography (CT) to quantitate 3D tumor volumes

We used serial micro CT scans to track lung tumor progression. We used ITK snap, a freely available software [36] to segment the entire tumor on consecutive 2D coronal slices which was then virtually stitched to provide tumor volume.

#### Serial micro CT

When acquiring micro CT, mice were anesthetized with isoflurane 1.5–2%, and then placed in the prone position with a respiratory sensor under the abdomen. Non-contrast-enhanced high-resolution micro CT scans with respiratory gating were performed (eXplore CT120 scanner, TriFoil Imaging, Chatsworth, CA) at a resolution of 100μm (100kV, 50mA). Two hundred and twenty views over 180° were acquired for each end-inspiration and end-expiration images. Total scan time per mouse was 11 minutes. Using manufacture measurements, typical dose for one whole body phenotyping scan is 3.5cGy x 5 scans which would be approximately 17.5cGy per mouse over 40 days. Images of the chest were reconstructed to a 100μm isotropic voxel.

#### Analysis of CT images

ITK snap was used by two experimenters to make volumetric measurements to track tumor progression (www.itksnap.org). All available consecutive 100μm thick coronal slices were used to manually segment lung tumors in a two-dimensional coronal plane carefully excluding heart, thymus, mediastinal lymph nodes and major blood vessels. This step was repeated along the z-axis to measure the entire lung tumor. Total tumor volume was then calculated using established methods [37] for each tumor mask.

### Detecting metastases in distant organs

Nine of the 11 CIH mice (2 mice died before harvest) and 12 of the 13 Sham mice (1 mouse died before harvest) were harvested at Day 40–41. Four of the 9 CIH mice and 5 of the 12 Sham mice underwent histological assessment of tumor in lungs, heart, and mediastinal lymph nodes.

#### Histological assessment

Lungs, heart and mediastinal lymph nodes underwent hematoxylin and eosin (H&E) staining to confirm CT scans for tumor size and location. In brief, mice were perfused with 0.9% normal saline and left/right lung, central mediastinal lymph nodes and heart were dissected en bloc and placed in formaldehyde for 48 hours, then cryopreserved with 30% sucrose for 5 days before being frozen and stored at -80°C. A cryostat was used to slice the tissue embedded in OCT at 40um and one slice in 6 was stained with H&E per manufacturer’s protocol. In addition, immunohistochemistry for Surfactant Protein C (EMD Millipore, AB3786) was performed to detect metastases within the heart. Micrographs were obtained using a 5-megapixel CCD Bayer Array RGB filter camera (DFC– 425, Leica, Germany) mounted on a digital light microscope (DM5500B, Leica, Germany) for H&E and DFC– 360FX camera for SPC.

### Statistical analysis

Continuous variables are presented as means and standard deviations. Categorical data are presented as frequencies and percentages. To evaluate the effect of CIH versus Sham on tumor volume progression, we used complementary strategies in the context of linear mixed models accounting for repeated micro CT scans in each mouse. First, we compared the linear slopes of tumor volume change between CIH and Sham by testing the significance of a group by ordinal time point variable interaction term. This test examines whether tumor volume changes at a faster/slower rate between the two experimental groups. In addition, to evaluate at which specific time points differences between CIH and Sham were observed, we used a repeated measures categorical data analysis to perform a joint hypothesis test evaluating the global null hypothesis of no differences in tumor volume between CIH and Sham at any of the measured time points (e.g., days 12, 19, 26, 33, 40). If this global null hypothesis was rejected, we examined the individual pairwise differences at each time point to determine when CIH and Sham differed. In addition to evaluating tumor progression, as an exploratory analysis we compared the overall variability of tumor volume between CIH and Sham mice within each time point, separately, using two-sided variance ratio tests.

Statistical significance was based on a p<0.05 in overall analyses or based on a Bonferroni-corrected p<0.01 in pairwise comparisons at each of the five time points post-injection, where applicable. Analyses were performed using Stata/SE 14.2 (StataCorp, College Station, TX), R software (www.r-project.org) or Microsoft Excel.

## Results

### Validation of intrathoracic injection and Cre-recombination activation

**Fig 1:**
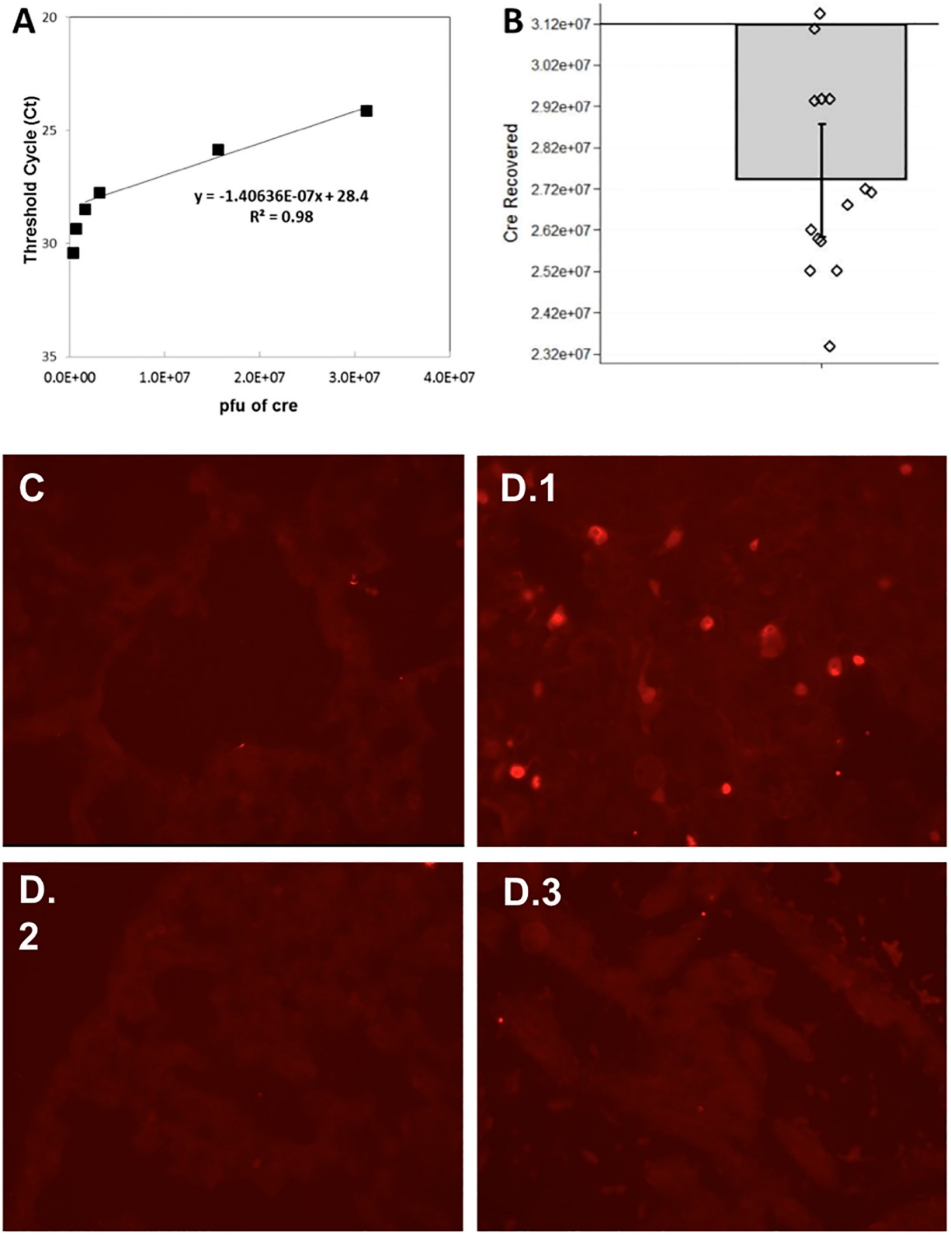
Validating intrathoracic injection technique. **(A)** A standard calibration curve was made using known quantities of Ad5CMVCre to then quantify unknown samples of Ad5CMVCre. Six different known concentrations of Ad5CMVCre were spiked into lung tissue to make the standard calibration curve: 3.12x10^5^, 6.24x10^5^, 1.56x10^6^, 3.12x10^6^, 1.56x10^7^, and 3.12x10^7^ pfu. **(B)** Amount of Ad5CMVCre (in pfu) measured in 14 mice that had their left lung injected with 3.12x10^7^ pfu Ad5CMVCre virus. Measurements were made using the TaqMan real-time PCR assay. The bar plot shows the mean with standard error and overlaying dot plot shows measurements of individual animals (n = 14). pfu = plaque-forming unit, a standard measure of the number of particles capable of forming plaques per unit volume. **(C-D)** To characterize Ad5CMVCre activity in the left lung of Kras^G12D+/-^; p53^fl/fl^; myristolated-p110α^fl/fl^-ROSA-gfp mice, gfp immunohistochemistry was used to detect Cre-recombination effect within the left lung, 4 days after Ad5CMVCre injection. **(C)** A representative micrograph of no gfp/Cre-recombination in the left lung of a mouse that had no Ad5CMVCre virus injected serves as a negative control. (**D.1**) A representative micrograph of activation of gfp/Cre-recombination in the left lung of a mouse that underwent Ad5CMVCre virus injection. To validate that the virus did not enter the bloodstream to turn on other primary cancers, (**D.2**) is representative of no gfp/Cre-recombination activation in the right lung and (**D.3**) is representative of no gfp/Cre-recombination activation in the heart.

To evaluate the reproducibility of intrathoracic injection of the Ad5CMVCre virus, we quantified the amount of Ad5CMVCre virus recovered from the left lung immediately after the injection using a standard calibration curve (Fig 1A). Among the 14 mice tested, thirteen mice fell within the standard curve region, while data from one mouse was slightly above the highest concentration of 3.12x10^7^ pfu (the amount of virus injected). The accuracy of our injection technique is validated by recovering nearly the entire amount of Ad5CMVCre virus; on average 88% of Ad5CMVCre was recovered (i.e., 2.74x10^7^ pfu compared to 3.12x10^7^ pfu injected; Fig 1B). Furthermore, our injection technique demonstrated consistency across animals, with a low coefficient of variation (CV%) of 8.4%.

To characterize Ad5CMVCre activity after injection into the left lung of Kras^G12D+/-^; p53^fl/fl^; myristolated-p110α^fl/fl^-ROSA-gfp mice, gfp immunohistochemistry was used to detect Cre-recombination effect within the left lung, 4 days after injection. Fig 1C is a micrograph representative of a negative control, the left lung of a mouse that had no Ad5CMVCre virus injected. Fig 1D.1 is a micrograph representative of positive gfp staining in a mouse that did have Ad5CMVCre virus injected into the left lung. Fig 1D.2 and 1D.3 are representative micrographs of the right lung and heart of the same mouse in Fig 1D.1. There is no gfp staining in the right lung and heart indicating no Ad5CMVCre migrated from the left lung.

### Effect of cyclical intermittent hypoxia on primary lung tumor progression

**Fig 2:**
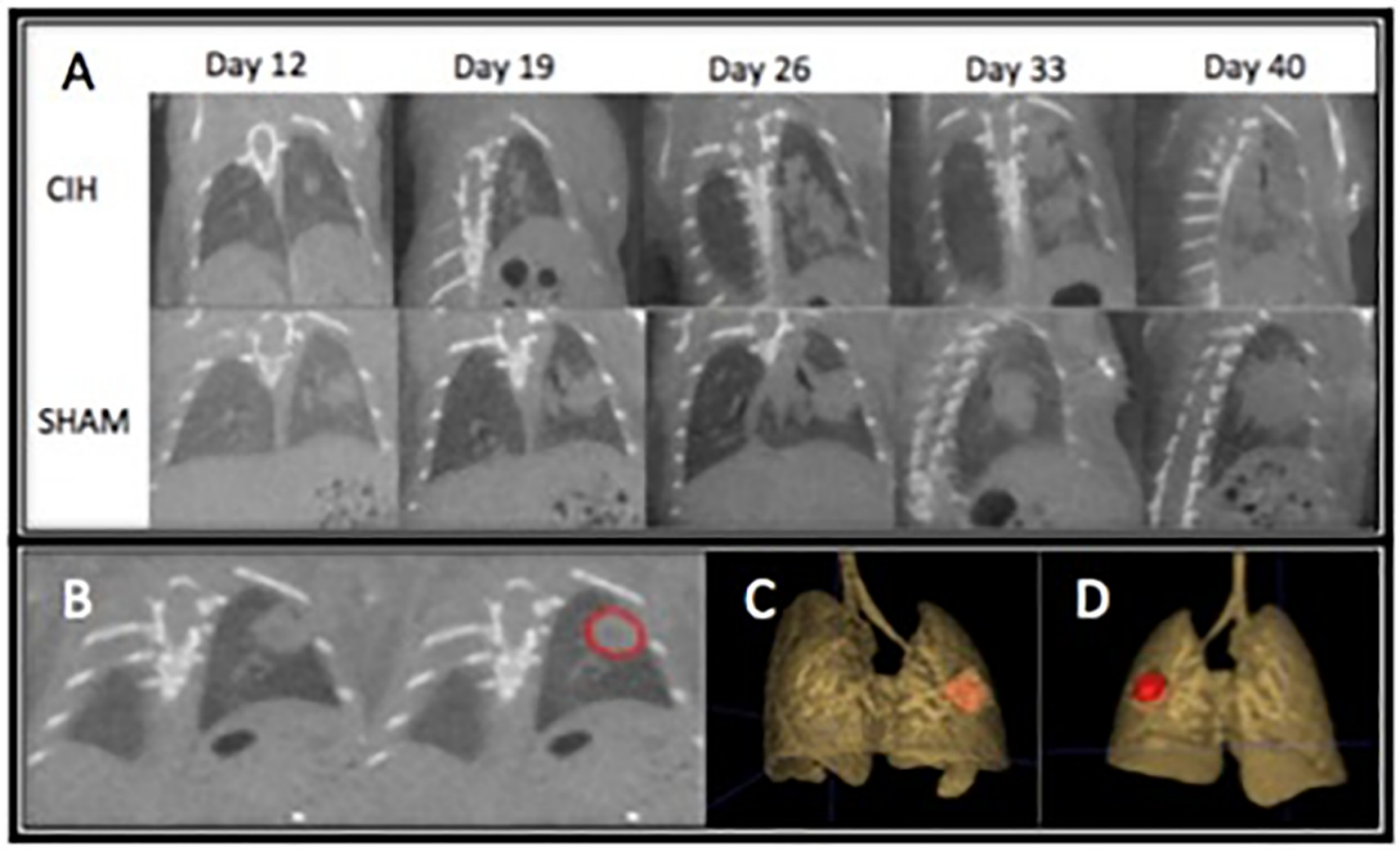
Representative micro CT of lung tumor progression. **(A)** Representative single 2D coronal slice (largest length x width) of serial micro CT scans of one mouse exposed to cyclical intermittent hypoxia (upper panel) and one mouse exposed to room air (Sham) (lower panel) at 12, 19, 26, 33, and 40 days after Ad5CMVCre intrathoracic injection. **(B)** Using ITK snap, manual segmentation of the lung tumor, outlined in red, allows for precise 2D contouring of lung tumors in consecutive 100um coronal slices. C & D. ITK snap then automatically stitches all 2D segmented tumors and calculates a 3D tumor volume that is then used to compare CIH vs Sham tumor progression. A representative 3D tumor anterior **(C)** and posterior **(D)** visualization of a lung tumor from the same mouse in **(B)** is colored in red.

**Fig 3:**
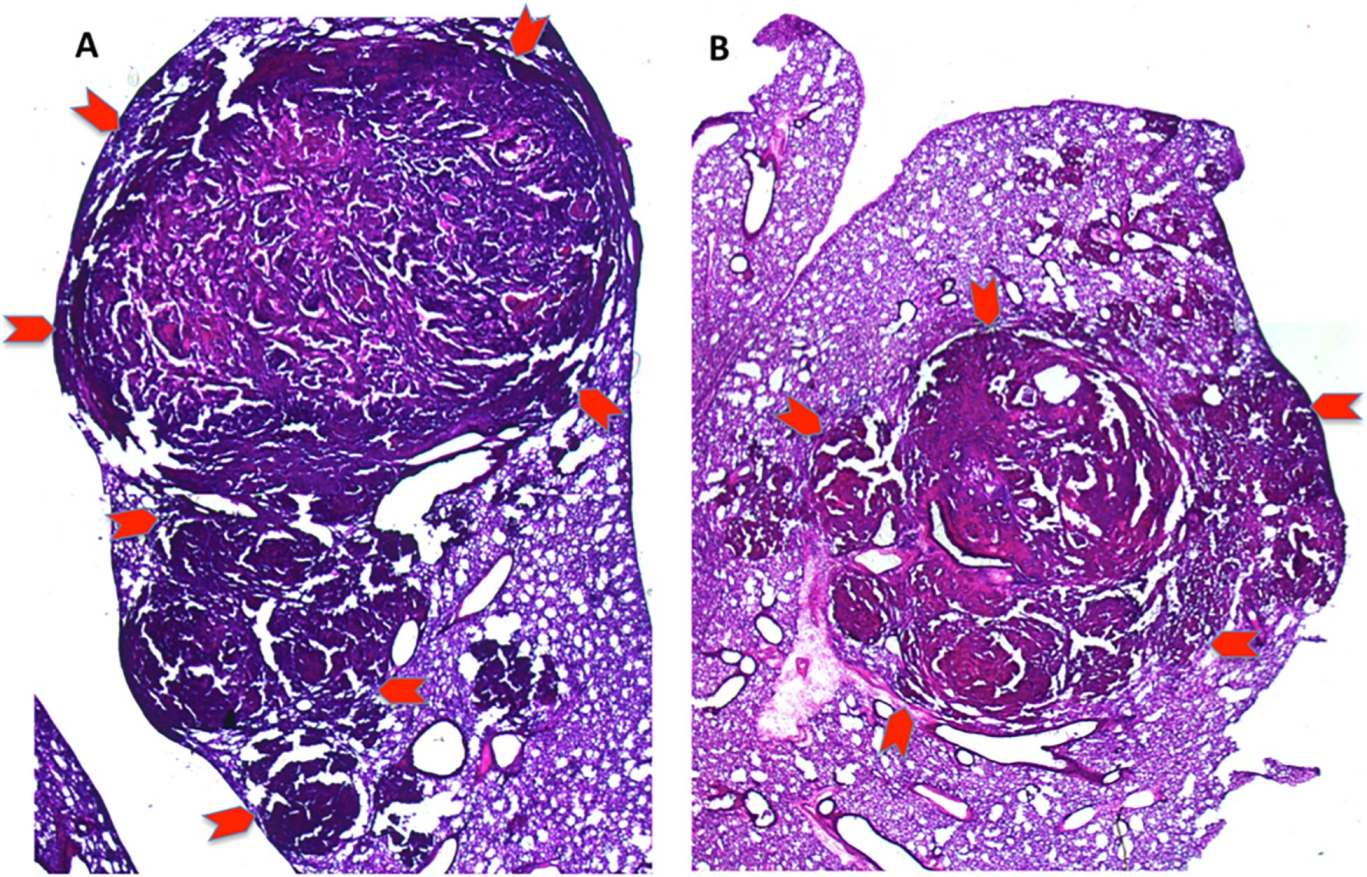
Representative H&E stain of left lung tumor of mouse exposed to cyclical intermittent hypoxia (A) compared to Sham (B). Primary tumor is outlined by red arrows [1.25X magnification].

Fast respiratory gating is critical to deal with artifact from a mouse’s respiration to accurately segment the tumor and while it has been available [38–40], it is increasingly more available in small animal micro CT software upgrades. Representative micro CT slices of increased tumor progression in mice exposed to CIH compared to Sham are shown in Fig 2A. Fig 2B is a representative visualization of how ITK snap was used to manually segment every consecutive 2D coronal slice to assess the primary tumor. All 2D segmented tumors was then virtually “stitched” to form and calculate a 3D tumor volume, with representative 3D tumors shown in Fig 2C (anterior view) and Fig 2D (posterior view). Left lung histology stained with H&E in CIH (Fig 3A) and Sham (Fig 3B) mice are similar to that reported by Sheen et al [17]. The H&E stained lung tumors were segmented using ImageJ to guide and confirm primary lung tumor volumes as seen on micro CT in mice exposed to CIH (n = 4) and Sham (n = 5). When tumor volumes were less than 100mm^3^ volumes between H&E and micro CT varied within 2-4mm^3^, and when tumor volumes were greater than 100mm^3^ differences ranged between 7-16mm^3^.

**Fig 4:**
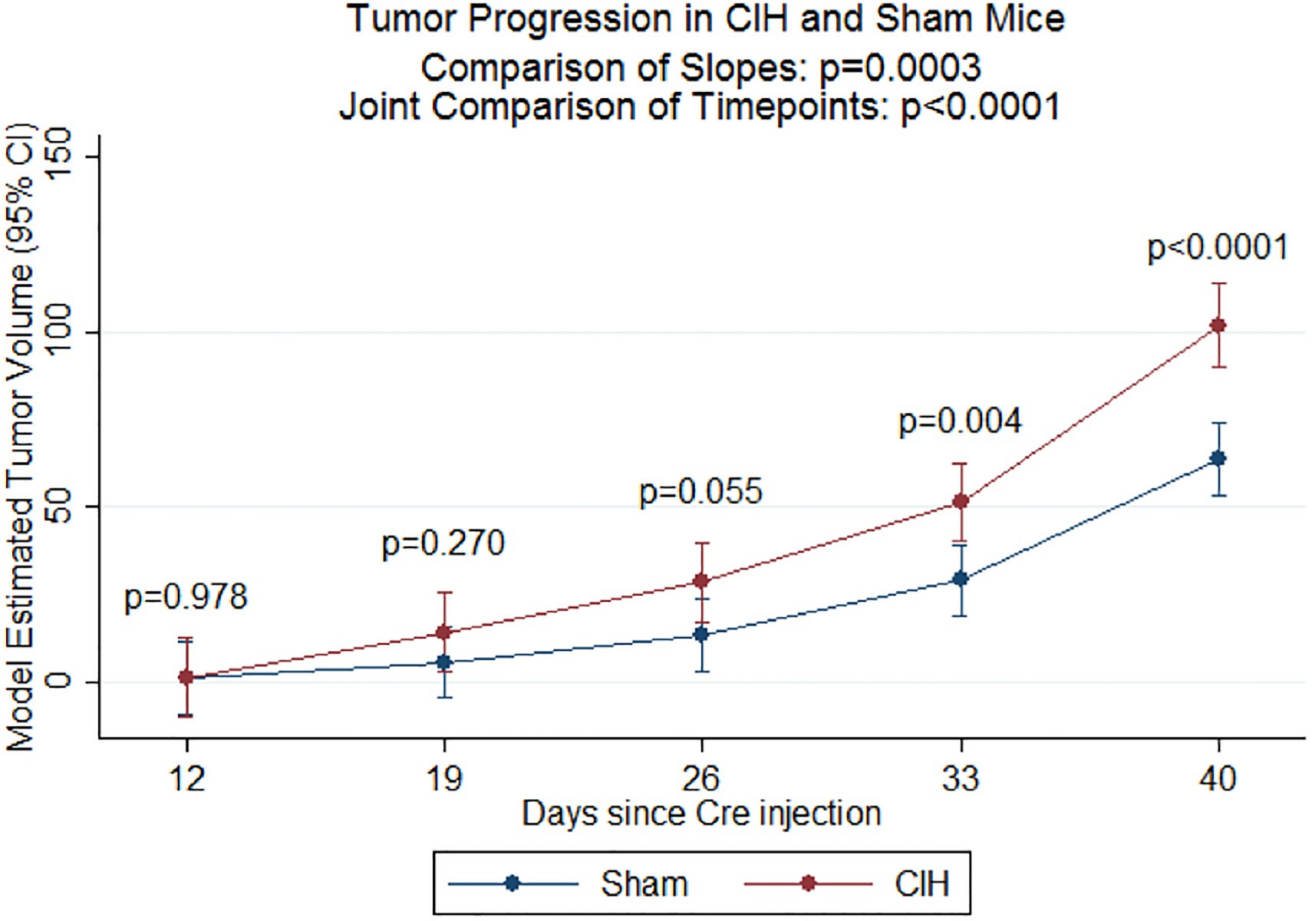
Tumor volume measured by micro CT scans. Tumor volume and associated 95% confidence intervals estimated from linear mixed model analysis are shown. Data for individual mice are shown. We found a significantly faster increase in tumor volume in the CIH versus Sham groups (p = 0.0003) and significant differences between the two groups across all timepoints (overall p<0.0001). In pairwise comparisons at each individual timepoint, the CIH group had trending or significantly greater tumor volume at day 26 (p = 0.055), day 33 (p = 0.004) and day 40 (p<0.0001). Sample size is as follows: for CIH at Day 12 (n = 11), Day 19 (n = 11), Day 26 (n = 11), Day 33 (n = 11), Day 40 (n = 10); for Sham at Day 12 (n = 13), Day 19 (n = 13), Day 26 (n = 13), Day 33 (n = 13), Day 40 (n = 13).

**Fig 5:**
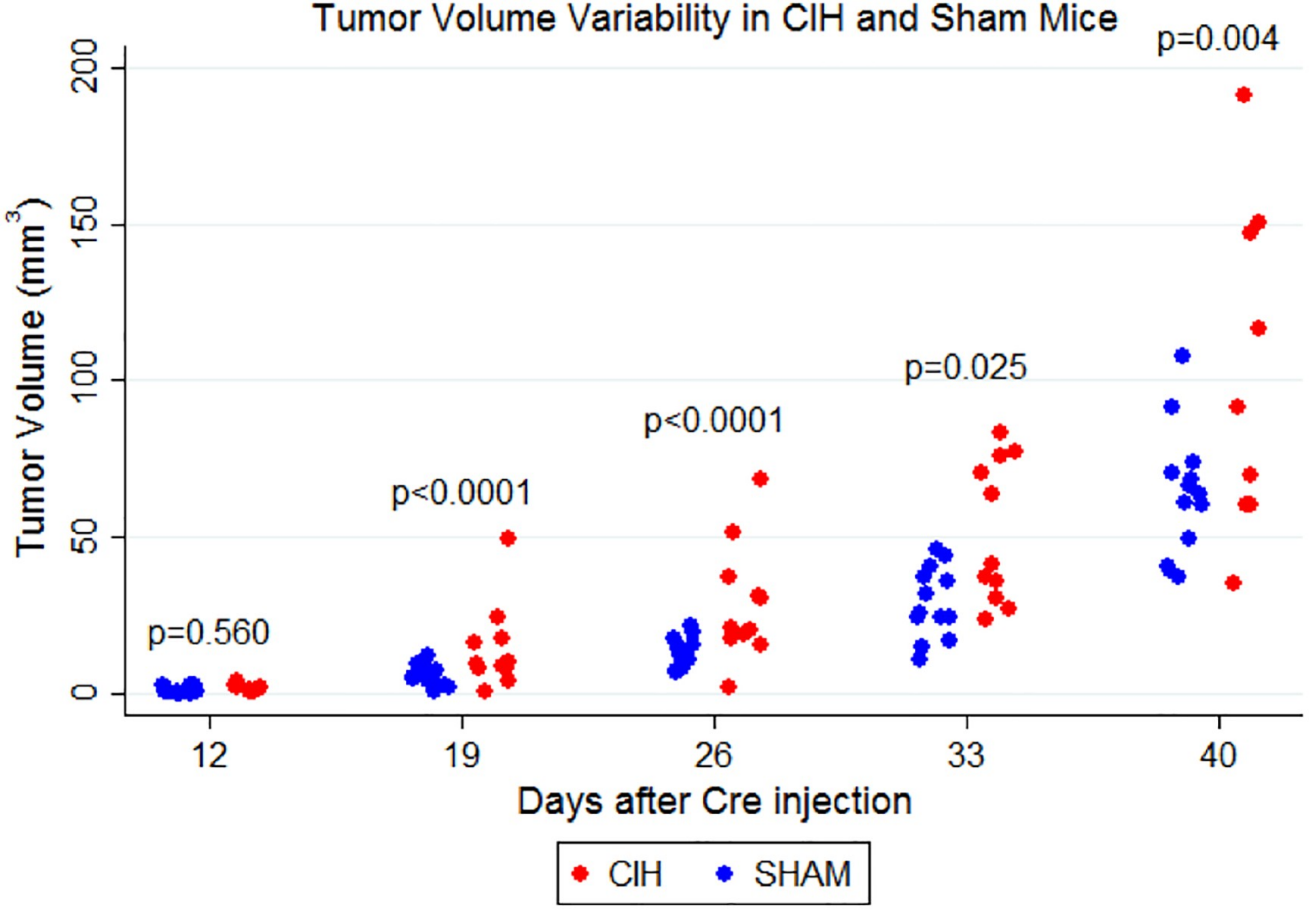
Variability in tumor volume as measured by micro CT scans. The measured tumor volume within the CIH and Sham groups in each time point is demonstrated. We found significantly greater variability in tumor volumes for the CIH group compared to Sham at day 19 (p<0.0001), day 26 (p<0.0001), day 33 (p = 0.025) and day 40 (p = 0.004).

Next, we examined the effect of exposing mice to CIH on tumor volume progression and variability. As demonstrated in Fig 4, we found that the CIH group had significantly faster increases in tumor volume compared to Sham (p = 0.0003 in comparison of slopes). Similarly, we were able to reject the global null hypothesis of no difference between CIH and Sham at any of the five timepoints (p<0.0001 in joint hypothesis test). In pairwise comparisons at each time point, we specifically observed significant differences in tumor volume at day 33 (p = 0.004) and day 40 (p<0.0001), based on our Bonferroni-corrected threshold of p<0.01, as well as suggestive evidence at day 25 (p = 0.055). As illustrated in Fig 5, there was nominal or significant evidence for increased variability in tumor volume in the CIH group at day 19 (p<0.0001; CV_Sham_ = 57.5%, CV_CIH_ = 93.9%), day 26 (p<0.0001; CV_Sham_ = 33.9%, CV_CIH_ = 65.4%), day 33 (p = 0.025; CV_Sham_ = 38.8%, CV_CIH_ = 44.2%,) and day 40 (p = 0.004; CV_Sham_ = 31.6%, CV_CIH_ = 50.9%).

### Distal metastases evident by time of harvest at Day 40–41

**Fig 6:**
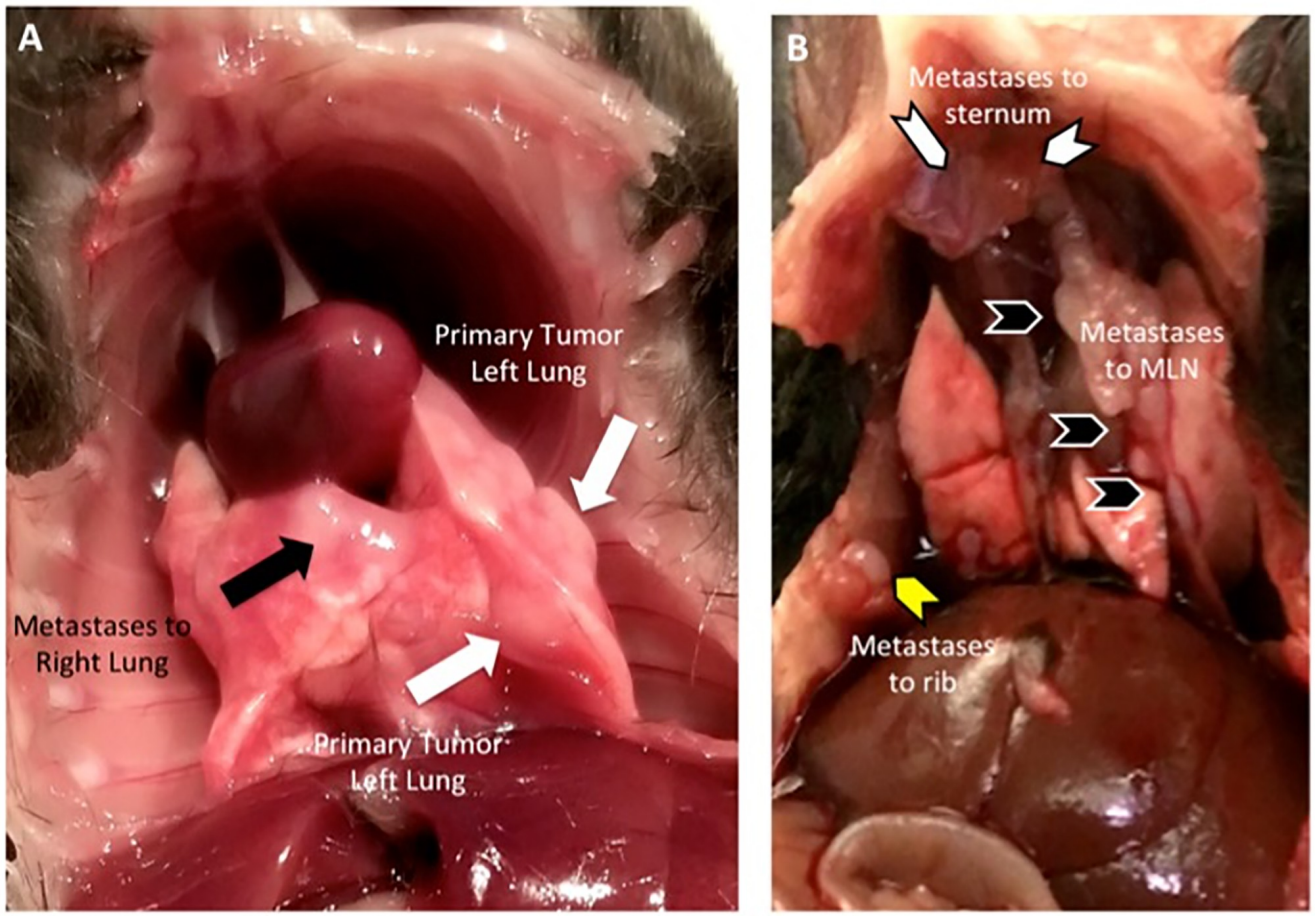
Metastases visualized at harvest. **(A)** Metastases to right lung (black arrows) is visualized at harvest in addition to primary tumor (white arrows) in the left lung (site of Ad5CMVCre virus injection). **(B)** Metastases to mediastinal lymph nodes (black chevron), rib (yellow chevron) and sternum (white chevron) is visualized at harvest.

At harvest on day 40–41 after Ad5CMVCre injection, in addition to visualizing primary tumor in left lung (Fig 6A, white arrows), we are able to visualize metastases to the right lung (Fig 6A, black arrows), mediastinal lymph nodes (Fig 6B, black chevron), ribs (Fig 6B, yellow chevron) and sternum (Fig 6B, white chevron).

**Fig 7:**
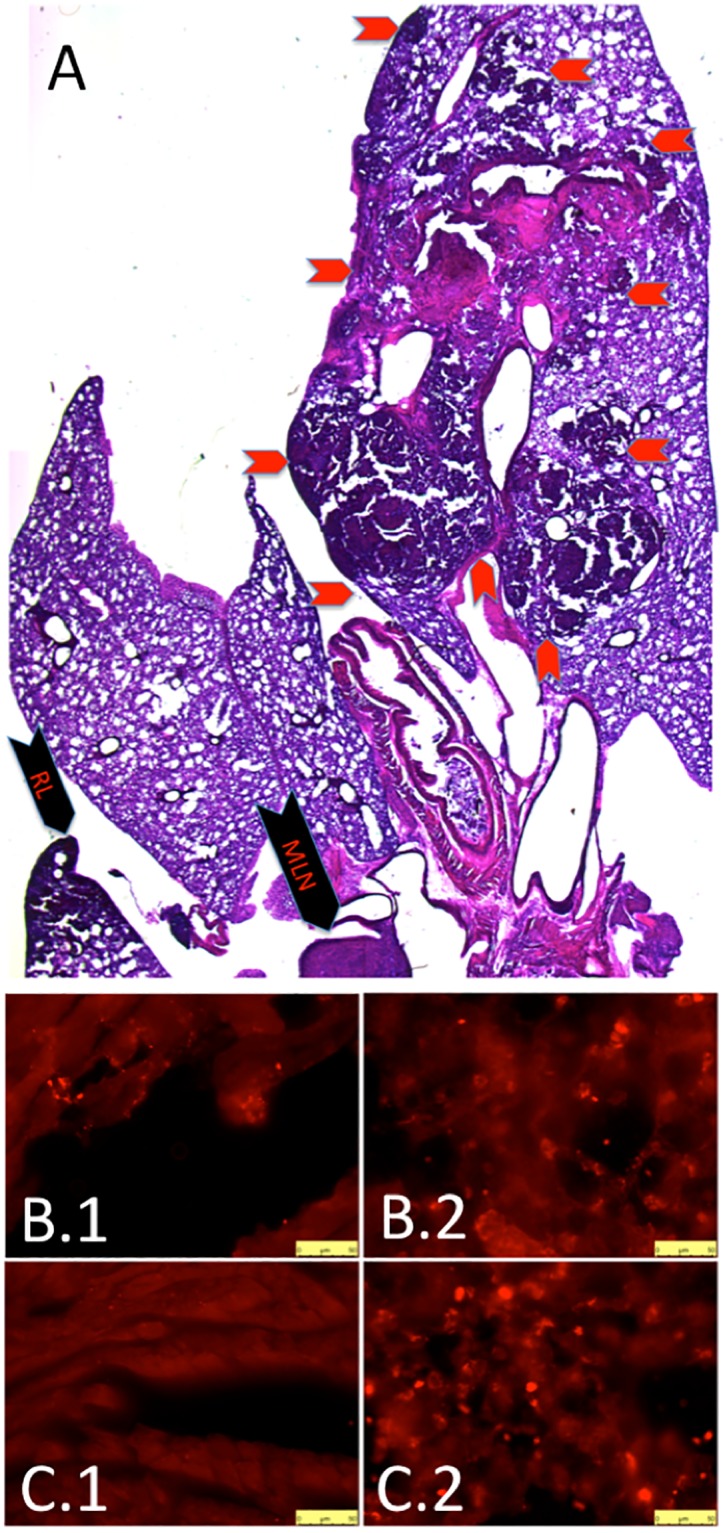
Metastases, histology. (**A**) H&E stain confirms primary tumor in left lung (red chevrons outline border of primary tumor) as well as metastases to right lung (black chevron-RL) and mediastinal lymph nodes (black chevron-MLN). Surfactant protein C (SPC) immunohistochemistry demonstrates metastases to the heart in a CIH mouse (**B.1**) and Sham mouse (**B.3**). We provide SPC immunohistochemistry to CIH lung (**B.2**) and Sham lung (**B.4**) for positive control. All micrographs taken at 40X, bar is 50 microns.

Using H&E stains, we confirmed left lung tumor (Fig 7A- red chevrons outline border of primary left lung tumor) and metastases to the right lung (Fig 7A- black chevron- indicating **“RL”**) and mediastinal lymph nodes (Fig 7A- black chevron- indicating **“MLN”**). In addition, we used SPC immunohistochemistry to confirm metastases to the heart in mice exposed to both CIH (Fig 7B.1) and Sham (Fig 7C.1). For positive control, we show SPC within the lung in both CIH (Fig 7B.2) and Sham (Fig 7C.2) mice.

## Discussion

To study the effect of CIH on lung cancer progression, we compared primary lung tumor volume and variability between mice exposed to sham (normoxia) or cyclical intermittent hypoxia. Mice exposed to CIH not only had faster tumor progression (as measured by micro CT), but also demonstrated increased variability in tumor size. Induction of a solitary lung tumor using an intrathoracic injection delivery of Ad5CMVCre virus within the Kras^G12D+/-^; p53^fl/fl^; myristolated-p110α^fl/fl^ mouse, resulted in metastases by Day 40–41 to distal organs including the right lung, heart, mediastinal lymph nodes, ribs and sternum.

### Cyclical intermittent hypoxia increases primary lung tumor progression and variability

When we exposed our GEM mice with lung cancer to CIH, mice had a significantly faster increase in tumor volume overall, with significant differences by Day 33 and 40 compared to Sham. To explain why CIH produces these effects, previous studies using a lung epithelial tumor model (where TC1 cells are injected into the flank of a mouse) revealed many important mechanisms for increased tumor progression with CIH [16, 41]. These include increased shift toward M2 protumoral phenotype in tumor-associated macrophages [16], a reduced granzyme B-producing CD3+CD8+GzmB+ T cells [41], increased circulating VEGF [42] and increased cancer stem cells markers on Oct4+ and CD44+CD133+ expressing TC1 tumor cells [41]. Given the significant role of oxygen in cancer progression [43], metastases [44–50] and targeted treatment [51, 52], it is critical that pre-clinical studies parallel the hypoxic environment of human cancers. While subcutaneous lung cancer models have the advantage of not requiring micro CT scans to track progression, this model is significantly more hypoxic than lung cancer in the lung [20]. Therefore, assessing these mechanisms will require further investigation in a GEM or syngeneic orthotopic lung model that better replicates the tumor microenvironment of the lung.

In addition, exposure to CIH increased variability in tumor size when compared to Sham animals. Given our high recovery rate of Cre virus, reproducibility of intrathoracic injections, and consistent occurrence of a solitary lung tumor it is unlikely that the CIH-specific increases in variability are related to the initial induction of tumor. Human non-small cell lung cancer is known to have a diverse set of pathological features with high heterogeneity [53]. Thus, it is conceivable that the addition of CIH alters the tumor microenvironment that leads to higher heterogeneity. This will require further investigation.

### Cyclical intermittent hypoxia does not show any differences in metastatic profile

We did not see any difference in the qualitative profile of metastases between CIH and Sham; both groups demonstrated metastases using H&E or SPC immunohistochemistry. It is possible that the aggressiveness of this model results in a ceiling effect with respect to metastases. Caswell et al [54] reported that a higher concentration of virus (via inhalation delivery) resulted in the mouse having significant tachypnea one month after tumor initiation (indicative of more intrapulmonary tumors), but had very few disseminated tumor cells in the pleural cavity (evidence of lymphatic metastases). They compared this aggressive model to one that used a lower concentration of virus where survival was 4–8 months (indicative of slower growing and fewer intrapulmonary tumors) and disseminated tumor cells were present with higher frequency, albeit in only half of the mice. Thus, results suggest that a careful balance between aggressiveness of the cancer model and maximizing survival time is required to study metastases in detail. Our method of turning on a solitary lung tumor using the highest concentration of Cre virus may be useful to study CIH-related effects on tumor initiation and tumor progression, but may not be useful to study CIH-related long-term effect on metastases. Future studies will need to optimize the concentration of Cre virus such that the cancer progresses at a slower rate allowing increased time for metastases to occur and develop.

### Significance of solitary lung tumor in GEM with normal lung tumor microenvironment: Comparison to other approaches

Transgenic mouse technology allows investigators to control inducible non-small cell lung carcinoma expression both spatially and temporally, in an immunocompetent background. A double mutation (Kras and p53 mutation) is the most common mouse lung cancer model, as these are the most common mutations in human lung cancer [24, 25]. Pre-clinical studies demonstrate that the Kras mutation has a significant role in lung cancer initiation but does not have a role in metastases [55, 56]. The p53 mutation has a significant role in cancer progression [57–59] but alone, rarely initiates lung adenocarcinomas [60] and is insufficient to drive metastases [54]. However, the combination of Kras and p53 missense mutations in transgenic mice (without Cre activation) results in spontaneous lung adenocarcinoma and metastases with a median survival of 266 days [61]. The addition of myristoylated-p110α mutation to Kras^G12D^ mutation and p53 deletion (with Cre activation) by Sheen et al [17] results in a faster lung tumor initiation, and facilitates metastatic potential. When allowed to reach an end point, Sheen et al [17] reports the mean survival of *heterozygous* myristoylated-p110α with Kras^G12D^ mutation and p53 deletion (Ad5CMVCre delivered using intratracheal instillation) to be 55 days. When we allowed mice to reach an end point using *homozygous* myristoylated-p110α with Kras^G12D^ mutation and p53 deletion (Ad5CMVCre delivered using intrathoracic injection), survival is about 40–45 days.

Staging human lung cancer is dependent on the measurement of a solitary lung tumor with any additional ipsi- or contra-lateral lung tumors considered to be metastases with a poor prognosis. To develop a more clinically relevant model, we employed an intrathoracic injection method [26] that results in a solitary primary lung tumor, which has advantages over intranasal inhalation [54, 62–64] and intratracheal instillation delivery of Ad5CMVCre virus [17, 65, 66]. Intranasal inhalation delivery of a non-specific Ad5CMVCre virus produces 87–539 adenomas [62] throughout the upper and lower respiratory track [60] 42 days post-infection with increasing conversion to adenocarcinoma by 112 days. Intratracheal instillation is more direct and consistently results in better reproducibility of virus delivery [64, 67], and depending on the GEM will produce fewer adenomas and lung carcinomas in 84 to 421 days [68]. While adenomas are benign, they still occupy space that obstructs ventilation in large [69] and smaller [70] airways leading to impairment of oxygen diffusion [71–74]. Subcutaneous tumor model (injection of cancer cells into the mouse’s flank) is another common approach to study cancer, but this approach has two disadvantages. First, Graves et al [20] demonstrated that this subcutaneous xenograft model of injecting human cancer cells into the flank of an immunosuppressed mouse, results in a tumor microenvironment that is significantly more hypoxic compared to orthotopic or spontaneous tumors in the lung. Because of significant levels of hypoxia in the lung vs. flank, they call into question conclusions made about mechanisms using subcutaneous tumor models. Second, injecting human cancer cells into an immunocompromised mouse hinders accurate study of immune surveillance within the lung tumor microenvironment [75]. Since our GEM was brought to a full C57BL/6 background with an intact immune system, future studies assessing the effect of CIH on the immune response and lung cancer progression/metastases will be more relevant.

### Strengths and limitations and future directions

This study has a number of strengths. Most importantly, the use of an intrathoracic technique results in a solitary lung carcinoma that is a better replication of human disease compared to intranasal inhalation and intratracheal instillation techniques. Another strength of this model is that the mice are immunocompetent on a C57BL/6 background, which allows a more comprehensive study of the potential immune related mechanisms by which CIH affects tumor progression and metastases. Collectively, this would allow for a more in-depth study of hypoxia inducible factors (Hifs) not only within the tumor microenvironment of the primary lung tumor and site of metastases, but also within cells of the immune system.

There are also limitations. Our technique utilized a nonspecific virus (Ad5CMVCre), which may result in non-lung cells becoming cancerous (e.g. adipocytes, fibroblasts, endothelial cells, etc.). To address this limitation, the Berns Lab has developed specific Cre viruses to initiate lung cancer specifically within the surfactant protein C (SPC) cell or the Clara cell antigen 10 (CC10) cell [65, 66] and are commercially available. Thus, a logical next step will be to combine the intrathoracic injection method with specific SPC or CC10 Cre viruses to ensure the cellular origin of tumors. Another limitation is that injection of the Cre virus directly into the lung may inadvertently allow the virus to enter the blood stream and produce false positive rather than true spontaneous metastases in distal organs. We addressed this by confirming virus activation of gfp in the left lung which was not present in the right lung or heart 4 days after Ad5CMVCre injection (a time point when virus is known to activate gfp [35] but is too early for metastases to develop), Thus, it can be inferred that using intrathoracic injection of virus into the left lung does not cause a primary tumor in the right lung and heart by viral migration. Furthermore, the higher rates of no tumor production and increased tumor variability in the CIH group could be due to the 2 weeks of CIH pre-treatment prior to Ad5CMVCre injection. While this pre-treatment exposure mirrors the clinical history that OSA most likely precedes lung cancer, CIH may alter the uptake and dynamics of the virus. In future studies we will compare effects of pre-treatment CIH vs. no pre-treatment CIH prior to Cre virus injection. Beyond limitations of the virus, this proof of concept study used only male mice, and there may be differences in cancer progression and metastases between male and female mice. We intend to study both genders in future studies with the more selective Cre viruses. Lastly, in this proof-of-concept study we employed the most severe CIH model, akin to an apnea hypopnea index of 60 events/hour, an oxygen desaturation index of 50, and a percentage of time under SaO2<90% of over 85%. Thus, while the severe CIH conditions we employed in this study are only relevant to a subset of patients with OSA, in the future we propose to test multiple other levels of CIH severity (mild and moderate) on tumor progression, metastases and recurrence using the improved Cre virus for induction of lung cancer.

## Conclusion

When evaluating the effect of cyclical intermittent hypoxia (CIH), mice exposed to CIH demonstrated increased tumor progression and variability compared to Sham controls, although there were no qualitative differences in metastatic profiles late in disease. We combined several previously described techniques to induce and track a solitary lung carcinoma in a GEM Kras^G12D+^; p53^fl/fl^; myristolated p110^fl/fl^ ROSA-gfp. This approach provides a more relevant tumor microenvironment within the lung to further study the role of the immune system and mechanisms by which CIH alters tumor progression and metastases in future analyses.

## Statement of translational relevance

Herein, we report that cyclical intermittent hypoxia, a pathological hallmark of obstructive Sleep Apnea (OSA) increases primary tumor progression and variability. Cyclical intermittent hypoxia may explain why some studies demonstrate an increased mortality of cancer when there is concurrent OSA. We introduce a clinically relevant preclinical mouse model of lung cancer, using a GEM with the ability to control spatial and temporal induction of solitary lung cancer that should facilitate the ability to make significant conclusions about tumor biology when translating into clinical practice.

## Supporting information

S1 File This is the raw data for Fig 4. (XLSX)

## Abbreviations

3D: Three dimensional
ANOVA: Analysis of variance
CC10: Clara Cell 10kD protein
cGy: centi Gray
CI: Confidence Interval
CIH: Cyclical Intermittent Hypoxia
CMV: Cytomegalovirus
CV: Coefficient of Variation
FiO_2_: Fraction of inspired oxygen
GEM: Genetically Engineered Mouse
gfp: green fluorescent protein
H&E: Hematoxylin and Eosin
Hif: Hypoxia inducible factors
ITK: Insight Segmentation and Registration Toolkit
Kras: Kirsten rat sarcoma
micro CT: micro computed tomography
NSCLC: Non-Small Cell Lung Cancer
OSA: Obstructive Sleep Apnea
p53: protein 53
p110α: protein 110-alpha
PaO_2_: partial pressure of arterial oxygen
RT-PCR: Reverse Transcription Polymerase Chain Reaction
SaO_2_: arterial oxygen saturation
SD: Standard Deviation
SPC: Surfactant Protein C
